# Multicenter trial of a perioperative protocol to reduce mortality in critically ill patients with peptic ulcer perforation: the PULP trial

**DOI:** 10.1186/cc9707

**Published:** 2011-03-11

**Authors:** M Hylander Møller

**Affiliations:** 1 University Hospital Bispebjerg, Copenhagen, Denmark

## Introduction

The aim of the present intervention study was to evaluate the effect of a multimodal and multidisciplinary perioperative care protocol on mortality in patients with peptic ulcer perforation (PPU). Sepsis is frequent and a leading cause of death in PPU patients, and morbidity and mortality is substantial [1,2].

## Methods

An externally controlled multicenter trial using historical and concurrent national controls in seven gastrointestinal departments in Denmark. Participants were 117 consecutive patients surgically treated for gastric or duodenal PPU between 1 January 2008 and 31 December 2009. The intervention was a multimodal and multidisciplinary perioperative care protocol based on the Surviving Sepsis Campaign. The main outcome measure was 30-day mortality.

## Results

**Figure 1:**
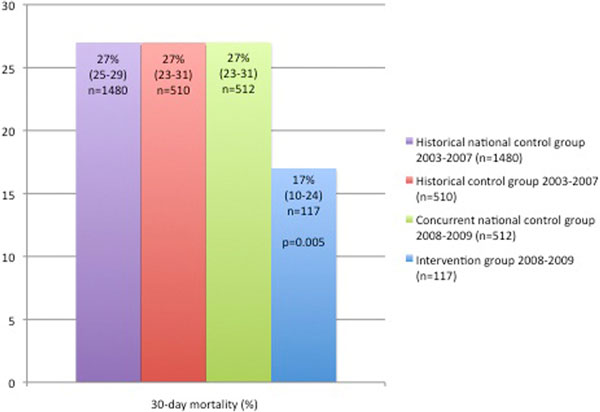
**Thirty-day mortality in the intervention group compared with the controls**.

Demographic characteristics were not different between the groups. The 30-day mortality proportion following PPU was 17% in the intervention group, compared with 27% in all three control groups; *P *= 0.005 (Figure 1). This corresponds to a relative risk (95% confidence interval) of 0.63 (0.41 to 0.97), a relative risk reduction of 37% (5 to 58) and a number needed to treat of 10 (6 to 38).

## Conclusions

The 30-day mortality in patients with PPU was reduced by more than one-third after the implementation of a multimodal and multidisciplinary perioperative care protocol based on the Surviving Sepsis Campaign, as compared with conventional treatment.
